# Equity of antiretroviral treatment use in high HIV burden countries: Analyses of data from nationally-representative surveys in Kenya and South Africa

**DOI:** 10.1371/journal.pone.0201899

**Published:** 2018-08-10

**Authors:** Sizulu Moyo, Peter W. Young, Eleanor Gouws, Inbarani Naidoo, Joyce Wamicwe, Irene Mukui, Kimberly Marsh, Ehimario U. Igumbor, Andrea A. Kim, Thomas Rehle

**Affiliations:** 1 HIV/AIDS, STIs and TB programme, Human Sciences Research Council, Cape Town, South Africa; 2 University of Cape Town, School of Public Health and Family Medicine, Cape Town, South Africa; 3 U.S. Centers for Disease Control and Prevention, Division of Global HIV & TB, Nairobi, Kenya; 4 The Joint United Nations Programme on HIV/AIDS (UNAIDS), Geneva, Switzerland; 5 Ministry of Health, National AIDS & STI Control Programme, Nairobi, Kenya; 6 U.S. Centers for Disease Control and Prevention, Division of Global HIV & TB, Pretoria, South Africa; 7 U.S. Centers for Disease Control and Prevention, Division of Global HIV & TB, Atlanta, Georgia, United States of America; National Institute of Health, ITALY

## Abstract

**Objective:**

To assess changes and equity in antiretroviral therapy (ART) use in Kenya and South Africa.

**Methods:**

We analysed national population-based household surveys conducted in Kenya and South Africa between 2007 and 2012 for factors associated with lack of ART use among people living with HIV (PLHIV) aged 15–64 years. We considered ART use to be inequitable if significant differences in use were found between groups of PLHIV (e.g. by sex).

**Findings:**

ART use among PLHIV increased from 29.3% (95% confidence interval [CI]: 22.8–35.8) to 42.5% (95%CI: 37.4–47.7) from 2007 to 2012 in Kenya and 17.4% (95%CI: 14.2–20.9) to 30.3% (95%CI: 27.2–33.6) from 2008 to 2012 in South Africa. In 2012, factors independently associated with lack of ART use among adult Kenyan PLHIV were rural residency (adjusted odds ratio [aOR] 1.98, 95%CI: 1.23–3.18), younger age (15–24 years: aOR 4.25, 95%CI: 1.7–10.63, and 25–34 years: aOR 5.16, 95%CI: 2.73–9.74 versus 50–64 years), nondisclosure of HIV status to most recent sex partner (aOR 2.41, 95%CI: 1.27–4.57) and recent recreational drug use (aOR 2.50, 95%CI: 1.09–5.77). Among South African PLHIV in 2012, lack of ART use was significantly associated with younger age (15–24 years: aOR 4.23, 95%CI: 2.56–6.70, and 25–34 years: aOR 2.84, 95%CI: 1.73–4.67, versus 50–64 years), employment status (aOR 1.61, 95%CI: 1.16–2.23 in students versus unemployed), and recent recreational drug use (aOR 4.56, 95%CI: 1.79–11.57).

**Conclusion:**

Although we found substantial increases in ART use in both countries over time, we identified areas needing improvement including among rural Kenyans, students in South Africa, and among young people and drug users in both countries.

## Introduction

Significant progress has been made in recent years in scaling up the provision of anti-retroviral therapy (ART) to people living with HIV (PLHIV) in sub-Saharan Africa. As of December 2015, over 17 million people globally were accessing ART, reaching the target of the *Treatment 2015* agenda established in 2012 by the Joint United Nations Programme on HIV/AIDS (UNAIDS), the World Health Organization (WHO), and partners, ahead of schedule [1,2].

Modelling suggests that high levels of ART coverage are required to control the HIV epidemic [3]. Despite progress in scaling up ART, only about half of all PLHIV in the East and Southern African region were receiving treatment by the end of 2015 [4]. This is partly due to global and country-specific recommended treatment guidelines that were in place in the past that recommended ART initiation until after onset of illness or immune suppression [5]). However, low ART coverage could also be explained by other structural factors such as health system deficiencies, individual factors such as lack of awareness of HIV status, healthcare seeking behaviours and risk behaviours, and socio-demographic factors [6]. Access to ART has been found to be greater in females than males, lower among young compared with older people [7–9], and among those living in poverty [10]. Stigma and discrimination [11,12], as well as incorrect or incomplete information about HIV and treatment have also been associated with lower ART use [13].

Identifying and eliminating barriers to access will be important if countries are to achieve universal ART coverage. WHO recommends assessing treatment equity and addressing obstacles to equitable access in line with the 2015 “treatment for all” recommendations which makes identifying and addressing potential barriers to achieving universal treatment coverage even more urgent [14]. For this study, we defined inequitable ART use based on differences in the proportion of PLHIV on treatment between groups defined by socio-economic, demographic, and behavioural characteristics.

Both Kenya and South Africa have been severely affected by the HIV epidemic. In Kenya HIV prevalence was estimated at 5.6% in 2012 with 1.4 million adult PLHIV and 898,000 people on ART by end of 2015 [15]. South Africa has the greatest number of PLHIV worldwide, estimated at 7 million in 2015, a 12.1% prevalence in the general population in 2012, and the largest ART programme in the world with approximately 3.4 million people on ART by end of 2015 [2].

Kenya and South Africa have undertaken nationally-representative population-based surveys with financial and technical support from the U.S. Centers for Disease Control and Prevention (CDC) to track both the burden of the epidemic in the general population as well as the impact of prevention and treatment strategies over time. These surveys have included biomarkers to assess HIV prevalence, HIV incidence and ART usage. Biomarker measurement of ART use is particularly useful since it is conducted on all HIV-positive samples and is not subject to self-reporting biases. We used these surveys to investigate differences in ART use among groups to shed light on whether equitable distribution of treatment has been achieved and maintained over time.

## Methods

### Survey design

Two Kenya AIDS Indicator Surveys (KAIS), implemented in 2007 and 2012, were conducted to measure behaviours and biomarkers related to HIV infection, including HIV status, recency of HIV infection, CD4+ T-cell count, HIV-1 ribonucleic acid levels, and ART status among PLHIV aged 15–64 years in 2007 and 18 months–64 years in 2012. In South Africa, the Human Sciences Research Council (HSRC) has conducted four cross-sectional nationally-representative surveys to assess HIV prevalence, incidence and related behaviours in the general population since 2002. Data from the 2008 and 2012 surveys are presented here. The methods and results of all of the surveys have been described elsewhere [16,17]. In summary, the surveys were designed using multi-stage cluster sampling of households from a master sample to generate representative HIV prevalence estimates by sex, locality type, region, and national domains (and by age and race in South Africa). For Kenya, locality type is defined dichotomously as urban or rural, while for South Africa there are four defined locality types namely: urban formal, urban informal (informal settlements in urban areas characterized by shanty towns); rural formal (farming areas); and rural informal (informal settlements in rural areas also characterized by shanty towns). The 2007 Kenya survey included respondents aged 15–64 years, while the 2012 survey included respondents aged 18 months–64 years. The South African surveys both included respondents of all ages.

In both the Kenyan and South African surveys, written informed consent was sought from respondents prior to household and individual interviews and blood collection; assent was obtained from children aged less than 18 years in addition to consent from their caregiver or guardian. In the South African surveys, institutional review boards provided a waiver to allow use of verbal consent if a respondent was not able to provide written consent. Venous whole blood was collected from participants and used to prepare dried blood spot (DBS) specimens in Kenya. During KAIS 2012, participants were also offered home-based counselling and testing with point-of-care CD4 testing offered to those who tested HIV-positive. In South Africa, DBS specimens were collected directly from capillary blood using a finger prick. In the 2007 Kenya and 2012 South African surveys, results of central HIV testing were made available for participant collection at a nearby health facility. Data from the questionnaires and the blood test results were linked using unique identifiers, which were anonymised prior to HIV and ART testing.

### Laboratory methods

Survey HIV prevalence was based on centralized testing of plasma (KAIS 2007) or DBS specimens (KAIS 2012; South Africa 2008 and 2012) tested with validated enzyme-linked immunosorbent assays based on national HIV testing algorithms, as previously described [16–18]. In Kenya, CD4+ T-cell counts were measured with BD FACSCalibur flow cytometer (Becton Dickinson BioSciences, San Jose, CA). Remaining plasma and DBS specimens were stored at -80°C for future testing.

For all surveys, a qualitative antiretroviral (ARV) drug assay was applied to test for a panel of ARVs defined separately for each country by liquid chromatography-tandem mass spectrometry in all HIV-positive specimens [19]. Frozen stored DBS specimens from Kenya were tested in 2015 while DBS specimens for the South African surveys were tested within two months of specimen collection. Drug panels used for each country were designed to cover one or more drugs in all approved first- and second-line regimens in use at the time of each survey (also including salvage regimens in South Africa). Panels used were nevirapine (NVP), efavirenz (EFV), lamivudine (3TC), and lopinavir (LPV) in Kenya and azidothymidine (AZT), NVP, and EFV, LPV, atazanavir (ATV) and darunavir (DRV) in South Africa. The ARV drug assay’s lower limit of detection was 0.02 μg/mL. Samples that fell above the lower limit of detection for any of the ARVs tested were classified as ARV positive.

### Data analysis

Data from each survey were analysed separately. Previously-developed survey weights were applied in order to provide estimates representative of the population from which each survey was drawn. Analysis was limited to individuals aged 15–64 years testing HIV-positive in centralized laboratory testing. North Eastern Province was excluded from the 2012 Kenya survey due to insecurity at the time of the survey and thus the province was also excluded from the 2007 survey analysis for consistency. In cells where the denominator is less than 25 the proportion and confidence intervals were replaced with an asterisk.

### Measures

The outcome measure of ART use was defined as having evidence of ART exposure in the survey blood specimen, i.e. a positive test for one or more ARVs among PLHIV. Note that treatment guidelines have evolved in both Kenya [20–22] and South Africa [23,24]. The guidelines in effect at the time of each survey are summarized in S1 Table.

A wealth index was created for each survey using measures related to household living standards including household characteristics (source of drinking water, access to electricity, main source of energy for cooking, type of toilet) and household ownership of assets (presence of a working refrigerator, radio, television, and in South Africa, mobile and landline phones) captured in the household questionnaire. The index was generated using factor analysis [25] and the resulting indices were grouped into quintiles from lowest (Quintile I) to highest (Quintile V) socioeconomic status (SES). As the indices were developed independently they are not directly comparable across countries.

Additional covariates were selected for inclusion in the analysis based on availability in the survey datasets and a structured literature review conducted prior to the survey that identified sex, age, disease stage, socio-economic status, employment, residency, cost, and decentralization of treatment as factors likely to predict ART use. Individual- and household-level factors identified in the review were included in the analysis.

For Kenya, employment status was defined as currently employed if the participant reported receiving payment or goods in exchange for work in the last seven days, otherwise they were unemployed. For South Africa, employment included self-employed as well as employment in the formal and informal sectors on a full-time or part-time basis. Students comprised those attending schools, including tertiary institutions.

### Statistical methods

Logistic regression analysis was performed to assess associations between lack of ART (no evidence of ART exposure in the blood specimen) and selected demographic, socioeconomic and behavioural variables separately for each survey. Significance of covariates for purposes of model building was assessed using Wald tests. Variables were selected for inclusion in initial models based on significance of association with ART use in bivariate analyses (p<0.1, presented in S3 and S4 Tables) or selected a priori in the case of sex, locality type, and household wealth. Models were then refined iteratively. Final regression models included covariates that were significantly associated with the outcome in multiple regression analysis (p<0.05) or that presented evidence of confounding as demonstrated by a 20% or greater change in coefficients of other significant covariates. Statistical significance for differences in prevalence of ART use between surveys was assessed by comparing confidence intervals (CI). For Kenya, separate models were developed including only women in order to further explore associations between pregnancy status and ART non-use.

To handle missing values, multiple imputation using chained equations was conducted with the Stata mi command on ART status, CD4 count, and disclosure of HIV status to last sex partner variables for each survey prior to regression analysis, to account for missing data for the Kenya surveys. The multiple imputation model assumes that data are missing at random. In South Africa, data were rarely missing and where this occurred the few cases affected were dropped at that stage of the analysis. A summary of the imputation modelling procedure is provided in S1 Text.

Analyses were conducted in SAS version 9.2 and Stata version 13 using commands designed to handle the complex survey design, except where otherwise noted.

### Ethical approval

The South African surveys were approved by the HSRC Research Ethics Committee and by the U.S. Centers for Disease Control and Prevention (CDC) in Atlanta, USA. A waiver of written consent was granted for situations where respondents were unable to provide written consent but were able to provide verbal consent with signature of an impartial witness. The Kenyan surveys were approved by the Ethical Review Committee of the Kenya Medical Research Institute and the Institutional Review Board of the CDC. The 2012 KAIS was also approved by the Committee on Human Research of the University of California, San Francisco.

## Results

**Fig 1:**
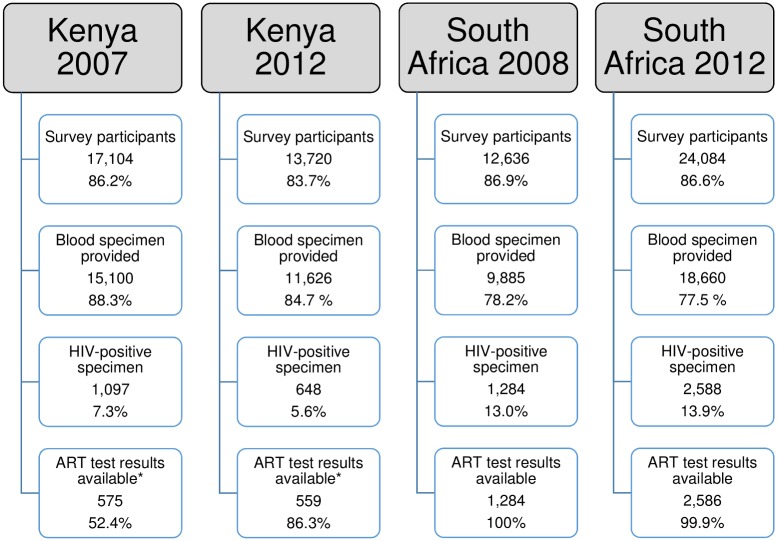
Study sample, HIV status and ART exposure testing, participants aged 15–64 years, Kenya and South Africa, 2007, 2008 and 2012. Notes: Data are unweighted. Percentages are calculated with respect to previous row, except interview response rates shown in first row which are calculated with respect to total eligible population (not shown). * For Kenya, 1092 cases have ART results available in 2007 and 635 have results available in 2012 after multiple imputation.

In Kenya, 17,104 persons aged 15–64 years participated in KAIS 2007, of whom 88.3% provided a blood specimen and 1,097 tested HIV-positive in the survey (Fig 1). Of these, 575 (52.4%) had ART testing results available for complete case analysis; a total of 1,092 cases were available after imputing missing values. In 2012, 13,720 persons aged 15–64 years participated in the study of which 11,626 (84.7%) provided blood specimens and 648 tested positive in the survey. Of these, 559 (86.3%) had an ARV test result available for analysis, while after imputing missing values a total of 635 cases were available. Additional information about the multiple imputation model is provided in S1 Text, including a table comparing the distribution of observed and completed values (S2 Table). In South Africa, 14,543 individuals participated in the 2008 survey, with 78.2% giving a blood specimen, and 1,284 testing HIV-positive (Fig 1). Corresponding figures for 2012 were 24,084 participants, 77.5% of whom gave a blood specimen, with 2,588 of these testing HIV-positive. Antiretroviral treatment results were available for almost all HIV-positive samples in South Africa (100% in 2008 [n = 1,284], and 99.9% [n = 2,586] in 2012) (Fig 1).

### Kenya

The proportion of PLHIV on ART increased from 29.3% (95% confidence interval [CI]: 22.8–35.8) in 2007 to 42.5% (95% CI: 37.4–47.7) in 2012. For females, it increased from 31.2% (95% CI: 23.0–39.4) to 45.6% (95% CI: 40.5–50.7). The unadjusted proportion on treatment also increased significantly for the Rift Valley region, for those aged 35–49 years, for persons who were married or cohabitating, and for those who disclosed their status to their most recent sex partner.

The proportion of PLHIV on ART did not differ significantly by locality, province or sex, education or household wealth quintile in either Kenyan survey. However, the likelihood of being on ART differed by age and marital status in both surveys. ART coverage differed significantly by current CD4 count in 2007 (p<0.001) but not in 2012. ART use varied by time since last HIV test in both surveys. In 2012, ART use varied by employment status (p = 0.021), disclosure to most recent partner (p = 0.046) and recreational drug use in the last 12 months (p<0.001). Women who were pregnant in the last three years were less likely to be on ART in both surveys (Table 1).

**Table 1:** ARV exposure by selected characteristics of HIV-infected individuals aged 15–64 years old, Kenya 2007 and 2012.

Variable	Kenya, 2007			Kenya, 2012		
	On ART			On ART		
	N	Weighted % (95% CI)	p-value	N	Weighted % (95% CI)	p-value
**Locality type**			0.875			0.240
Rural	389	29.7 (22.3–37.1)		317	39.9 (32.5–47.2)	
Urban	186	28.5 (15.5–41.4)		242	45.9 (38.9–52.9)	
**Province**			0.219			0.240
Nairobi	79	36 (14.3–57.7)		54	45.1 (32.4–57.8)	
Central	41	40.2 (25.3–55.2)		50	47.6 (34.2–61.0)	
Coast	78	15.4 (3.1–27.8)		55	41 (23.7–58.3)	
Eastern	47	28.9 (4.3–53.4)		66	59.3 (41.3–77.2)	
Nyanza	197	35.6 (22.2–49.0)		212	36.6 (29.1–44.0)	
Rift Valley	77	17.6 (8.9–26.3)		70	45.5 (29.4–61.6)	
Western	56	28.9 (10.5–47.3)		52	36.8 (22.7–50.8)	
**Sex**			0.259			0.120
Male	203	25.7 (18.5–32.9)		175	37.8 (28.6–46.9)	
Female	372	31.2 (23.0–39.4)		384	45.6 (40.5–50.7)	
**Age (years)**			< .001			< .001
15–24	100	18.2 (8.3–28.1)		59	21.6 (8.8–34.3)	
25–34	212	23 (15.0–31.0)		182	25.1 (18.1–32.2)	
35–49	208	38.3 (29.6–47.1)		229	55 (47.7–62.4)	
50–64	55	38.2 (24.2–52.3)		89	59.6 (48.6–70.6)	
**Marital status**			0.002			0.002
Single/never married	70	22.8 (10.1–35.6)		73	24.5 (12.8–36.2)	
Married/cohabitating	373	25.5 (19.3–31.7)		337	42 (35.4–48.7)	
Divorced/separated/widowed	132	42 (30.6–53.3)		149	53.1 (44.7–61.5)	
**Education**			0.943			0.130
None	292	30 (20.7–39.3)		81	35.5 (24.0–46.9)	
Primary	181	27.4 (19.9–35.0)		298	39.5 (32.4–46.6)	
Secondary	77	29.5 (19.7–39.4)		28	45.7 (25.7–65.8)	
Higher	25	32.7 (9.8–55.6)		151	50.6 (40.8–60.4)	
**Household wealth**			0.507			0.900
Quintile I (lowest)	98	27.5 (14.7–40.2)		87	41 (28.0–54.1)	
Quintile II	102	20.7 (8.5–32.9)		133	40.6 (28.8–52.3)	
Quintile III	96	31.9 (19.3–44.5)		122	41.2 (30.9–51.4)	
Quintile IV	123	31.7 (19.8–43.7)		137	42.7 (33.2–52.1)	
Quintile V (highest)	156	33.2 (24.2–42.3)		80	48.6 (34.9–62.3)	
**Employment**			0.432			0.021
unemployed	90	33.7 (19.4–48.1)		197	50 (41.1–58.8)	
employed	485	28.5 (22.1–34.9)		361	39 (33.5–44.5)	
**CD4 category (cells/mm**^**3**^**)**			< .001			0.310
< = 250	106	24.1 (15.4–32.8)		44	51.5 (37.0–65.9)	
250>-350	60	53.4 (38.1–68.8)		35	59.9 (42.5–77.4)	
350>-500	93	32.9 (22.2–43.7)		37	47.6 (27.9–67.4)	
>500	276	23.9 (15.6–32.2)		168	42.4 (32.9–51.9)	
**Most recent HIV test**†			< .001			< .001
<12 months ago	135	38.1 (27.5–48.7)		264	49.9 (42.1–57.6)	
1–2 years ago	69	47.1 (32.8–61.5)		100	32.9 (21.7–44.1)	
> 2 years ago	84	41.3 (25.4–57.2)		122	51.7 (42.1–61.3)	
**Disclosed results to partner**‡			0.572			0.046
Yes	193	35.3 (25.8–44.8)		174	69 (60.9–77.0)	
No	38	27.9 (5.3–50.4)		90	55 (43.1–66.9)	
**Pregnancy and HIV testing**§						
Pregnant	141	18.5 (10.3–26.7)	< .001	98	35.2 (24.6–45.8)	0.031
Not pregnant	231	38.1 (28.5–47.8)		286	49.1 (43.2–55.0)	
Antenatal care (ANC) visit	123	18.8 (10.1–27.5)	0.727	95	35.2 (24.5–45.9)	0.988
No ANC visit	18	*		3	*	
Ever tested for HIV in pregnancy	76	22.3 (11.7–32.9)	0.172	88	33.3 (22.0–44.6)	0.288
Never tested for HIV in pregnancy	47	13.4 (2.9–23.9)		7	*	
**Recreational drug use**§§						< .001
Yes	-			55	17.9 (6.5–29.4)	
No				504	45.3 (40.2–50.5)	
**Total**	575	29.3 (22.8–35.8)		559	42.5 (37.4–47.7)	

Notes: All numbers are unweighted except where otherwise specified.

* Suppressed due to denominator <25 observations.

^**†**^ Applies to respondents who reported ever testing for HIV

^‡^ Applies to respondents who self-reported HIV-positive, partner refers to most recent sex partner.

^§^ Applies to pregnancy during last 3 years among female respondents only.

^§§^ Drug use in last 12 months.

ART = antiretroviral treatment, CI = confidence interval.

In bivariate analysis, in 2012, ART non-use was also greater among women aged 15–24 years (OR = 5.6) and 25–44 years (OR = 4.09) compared with those aged 50–64 years (p<0.001), and inversely with being married or cohabitating, or divorced, separated or widowed, compared to those who were single/never married (p<0.001), having last HIV test 1–2 years ago versus <1 year ago, not disclosing HIV status to their most recent sex partner (p = 0.011), pregnancy in the last three years among women (p = 0.045) and reporting having used recreational drugs in the last 12 months (p = 0.003) (S3 Table).

In multiple regression, only younger age was associated with lack of ART use among PLHIV in 2007, with an adjusted odds ratio (aOR) of ART use of 3.69 (95% CI: 1.55–8.79) for PLHIV aged 15–24 versus 50–64 years of age. In 2012, when controlling for residency, age group, disclosure, and recreational drug use, ART non-use was more likely in respondents living in rural compared with urban areas (aOR 1.98, CI 1.23–3.18), and in younger respondents (aged 15–24 years, aOR 4.25, CI 1.70–10.63, aged 25–34, aOR 5.16, CI 2.73–9.74 compared with those aged 50–64 years). ART non-use was also more likely in those who reported they had not disclosed their status to their last sex partner (aOR 2.41, 95% CI: 1.27–4.57) and those who reported a recreational drug use in last 12 months (aOR 2.50, 95% CI: 1.09–5.77) (Table 2). In order to explore the association between recent pregnancy and ART non-use among women, separate multiple regression models were constructed for women in both 2007 and 2012. No significant association between lack of ART use among those recently pregnant was found in 2007 (aOR 1.30, 95% CI: 0.76–2.21) or 2012 (aOR 0.97, 95% CI: 0.54–1.74). However, the women-only model for 2012 did reveal associations with marital status, education, and wealth quintile not seen in the combined male-female model. Lack of ART was significantly less likely among married women compared with single women (aOR 0.26, 95% CI: 0.12–0.58), as among women who were divorced, separated or widowed (aOR 0.32, 95% CI: 014–0.73). Wealth quintile also showed a 22% increase in odds of ART use for each increase in wealth quintile (aOR of non-use: 0.78, 95% CI: 0.64–0.95). Unlike in the combined male-female model, disclosure of status and recreational drug use in last 12 months was not found to be associated with ART non-use in women (S5 Table).

**Table 2:** Multiple regression analysis showing associations between selected characteristics of HIV-infected individuals with ART non-use among individuals aged 15–64 years, Kenya, 2007 and 2012.

	Kenya, 2007						Kenya, 2012					
	All PLHIV (n = 1097)			Female PLHIV (n = 729)			All PLHIV (n = 648)			Female PLHIV (n = 454)		
Variable	aOR	95% CI	p-value	aOR	95% CI	p-value	aOR	95% CI	p-value	aOR	95% CI	p-value
**Residence**												
Rural							1.98	(1.23–3.18)	0.005			
Urban (ref)												
**Age (years)**												
15–24	3.69	(1.55–8.79)	0.004	3.30	(1.07–10.22)	0.039	4.25	(1.7–10.63)	0.002	9.21	(3.13–27.12)	<0.001
25–34	2.18	(0.96–4.95)	0.061	1.86	(0.66–5.28)	0.235	5.16	(2.73–9.74)	<0.001	5.30	(2.44–11.52)	<0.001
35–49	1.31	(0.68–2.51)	0.419	1.13	(0.47–2.70)	0.783	1.48	(0.85–2.56)	0.165	1.62	(0.78–3.34)	0.194
50–64 (ref)												
**Marital status**												
Single/never married (ref)												
Married/cohabitating										0.26	(0.12–0.58)	0.0011
Divorced/separated/widowed										0.32	(0.14–0.73)	0.0069
**Education**												
None (ref)												
Primary										0.79	(0.36–1.71)	0.5419
Secondary										1.00	(0.26–3.85)	0.9962
Higher										0.41	(0.19–0.89)	0.0250
Wealth quintile*										0.78	(0.64–0.95)	0.0122
**Pregnancy in last 3 years**												
Yes				1.30	(0.76–2.21)	0.338				0.97	(0.54–1.74)	0.9291
No (ref)												
**Disclosed HIV status to most recent partner**†												
Yes (ref)												
No							2.41	(1.27–4.57)	0.007			
Unaware of status							9.15	(5.19–16.13)	<0.001			
**Recreational drug use in last 12 months**												
No (ref)												
Yes							2.50	(1.09–5.77)	0.032			

Notes: Although current CD4 category was significantly associated with ART status in bivariate analysis, as a marker of current immune status it is likely to be confounded by duration of treatment, so it was not included in the regression analysis.

* Odds of ART non-use was found to decrease linearly with wealth quintile among women in 2012 so it is included as a continuous term.

^†^ Applies to respondents who self-reported HIV-positive.

### South Africa

Overall the proportion of PLHIV on ART increased from 17.3% (95% CI: 14.2–20.9) in 2008, to 30.3% (95% CI: 27.2–33.6) in 2012. ART use increased more than four-fold among those living in rural informal areas from 7.7% (95% CI: 3.7–15.4) in 2008 to 34.3% (95% CI: 30.8–38.9) in 2012 (S1 Fig), and more than doubled among females from 16.8% (95% CI: 13.5–20.7) in 2008 to 43.2% (95% CI: 30.9–37.7) over the same period. Although ART use increased across all age groups, increases were much greater among the older age groups with 14.9% (95% CI: 8.9–23.7) of those aged 50–64 years on ART in 2008 compared with 43.3% (95% CI: 36.1–50.7) in 2012. The proportion of PLHIV on ART differed significantly by marital status in both survey years (p = 0.031 in 2008 and p = 0.002 in 2012). In 2012 the proportion of PLHIV on ART also differed significantly by sex (p = 0.001), by age (p<0.001), by employment status (p<0.001) and by recreational drug use (p<0.0001) (Table 3). On bivariate analysis, ART non-use was significantly associated with province, marital status, highest level of education, employment status and household wealth in 2008. In 2012 there was significantly greater ART non-use among males (p = 0.001), those aged 15–24 (OR 4.57, p<0.001) and 25–34 years (OR 2.75, p<0.001) versus those aged 50–64 years, those who were employed (p<0.001) or students (p<0.033) versus unemployed, and those with recent recreational drug use (p<0.001), while those who were divorced/separated/widowed had lower non-use of ART compared with those who were single/never married/no stable partnership (p<0.017). Recency of testing showed no significant association with ART non-use in either survey year (S4 Table).

**Table 3:** Antiretroviral exposure by selected characteristics of HIV-infected individuals aged 15–64 years old, South Africa, 2008 and 2012.

Variable	South Africa, 2008			South Africa, 2012		
	ART			On ART		
	N	Weighted % (95% CI)	p-value	N	Weighted % (95% CI)	p-value
**Locality type**			0.211			0.068
Urban formal	481	15.9 (11.3–22.0)		888	27.1 (21.3–33.8)	
Urban informal	345	20.5 (14.1–29.0)		525	27.2 (22.7–32.3)	
Rural formal	117	19.4 (13.9–26.2)		335	27.5 (19.6–37.2)	
Rural informal	341	7.7 (3.7–15.4)		840	34.7 (30.8–38.9)	
**Province**			0.710			0.200
Western Cape	83	28.3 (16.1–44.7)		157	27.5 (18.6–38.6)	
Eastern Cape	146	19.1(10.1–33.2)		324	31.2 (25.1–38.2)	
Northern Cape	70	18.9 (8.9–35.8)		131	13.6 (5.6–29.6)	
Free State	125	13.5 (8.1–21.6)		258	27.0 (20.2–35.1)	
KwaZulu Natal	288	15.4 (10.2–22.6)		726	32.8 (28.2–37.7)	
Northwest	114	21.5 (13.8–31.8)		211	39.6 (30.0–50.2)	
Gauteng	211	18.6 ([10.7–30.4)		291	26.2 (18.0–36.5)	
Mpumalanga	141	16.4 ([10.7–24.2)		291	35.9 (27.9–44.7)	
Limpopo	106	13.8 ([7.3–24.7)		197	24.8 (15.6–37.1)	
**Race**			0.864			0.102
Black African	1205	17.3 (14.2–21.0)		2365	30.0 (26.8–33.4)	
Other race	79	16.2 (7.6–31.5)		223	40.8 (29.0–53.7)	
**Sex**			0.650			0.001
Male	352	18.5 (12.6–26.4)		801	24.1 (19.5–29.5)	
Female	932	16.8 (13.5–20.7)		1787	43.2 (30.9–37.7)	
**Age (years)**			0.350			<0.001
15–24	279	11.6 (4.9–25.2)		399	14.3 (10.0–20.0)	
25–34	441	17.7 (13.0–23.6)		926	21.7 (17.9–26.1)	
35–49	444	20.8 (15.9–26.8)		912	41.0 (35.5–46.7)	
50–64	120	14.9 (8.9–23.7)		349	43.3 (36.1–50.7)	
**Education**			0.177			0.661
None	73	6.5 (2.2–17.5)		-	-	
Primary	297	16.0 (10.7–23.3)		568	32.8 (27.4–38.8)	
Secondary	794	17.5 (13.6–22.3)		1544	30.4 (26.7–34.4)	
Higher education	43	31.1 (12.5–58.7)		84	26.5 (13.9–44.6)	
Missing/unknown*	77	-		392	-	
**Marital status**			0.031			0.002
Single	726	17.5 (13.2–23.0)		530	33.3 (26.0–41.6)	
Married	342	13.2 (9.3–18.4)		1784	27.7 (24.4–31.3)	
Divorced/separated/widowed	143	28.8 (19.3–40.5)		229	47.0 (38.8–55.3)	
**Household wealth**			0.118			0.738
Quintile I	375	15.1 (10.6–21.2)		759	31.4 (26.9–36.4)	
Quintile II	382	15.0 (10.9–20.4)		683	29.1 (24.1–34.6)	
Quintile III	243	24.5 (16.7–34.4)		652	31.1 (24.2–38.9)	
Quintile IV	197	13.1 (7.7–21.5)		315	27.0 (19.4–36.4)	
Quintile V	68	24.0 (9.8–47.8)		148	37.8 (24.2–53.6)	
**Employment**			0.186			<0.001
Unemployed	706	20.4 (16.2–25.5)		1489	33.9 (30.2–37.9)	
Employed	426	11.4 (8.1–15.7)		101	9.7 (4.2–20.9)	
Student	61	22.2 (5.2–59.6)		933	27.8 (23.2–32.8)	
**Most recent HIV test**			0.328			0.209
<12 months	365	21.8 (15.6–29.4)		1276	35.4 (31.3–39.7)	
1–2 years	192	17.3 (10.9–26.5)		266	33.4 (25.8–42.0)	
2–3 years	91	23.4 (13.2–37.9)		152	30.5 (21.8–40.9)	
3+ years	98	31.3 (19.5–46.0)		245	44.3 (33.6–55.6)	
**Alcohol intake in last 12 months**			0.491			0.066
Non-high-risk drinker	1083	16.6 (13.3–20.6)		2050	31.5 (27.9–35.3)	
High-risk drinker	113	20.8 (11.1–35.7)		249	21.2 (13.4–31.8)	
**Recreational drug use**†			0.871			<0.001
Non-drug user	10101	17.5 (14.0–21.5)		2345	32.1 (28.9–35.6)	
Drug user	45	15.8 (4.1–45.1)		122	9.0 (4.3–17.6)	
**Total**	1284	17.4 (14.2–20.9)		2586	30.3 (27.2–33.6)	

Notes: All numbers are unweighted except where otherwise specified.

* Estimates not presented for missing/unknown categories.

^†^ Refers to last 3 months.

On multiple regression analysis, marital status was significantly associated ART non-use: being divorced, separated or widowed was associated with a lower likelihood of ART non-use when compared to being single or never married (aOR 0.45, 95% CI: 0.22–0.01, p = 0.027). Significant associations were also observed by level of education (aOR 0.14, 95% CI: 0.03–0.68, p = 0.014 for tertiary (higher) level of education versus no education), employment status (aOR 2.16, 95% CI: 1.26–1.37, p = 0.01 for those who were employed versus the unemployed) and household wealth (aOR 0.43, 95% CI: 0.20–0.90, p = 0.03 for Quintile III versus Quintile I) in 2008. In 2012, younger age was significantly associated with ART non-use (aOR 4.23, 95% CI: 2.56–6.70, p<0.001 and aOR 2.84, 95% CI: 1.73–4.67, p<0.001 for age groups 15–24 years and 25–34 years, respectively, compared to age group 50–64 years). Students were significantly more likely not to use ART (aOR 1.61, 95% CI: 1.16–2.23) compared to those who were unemployed. Recreational drug users were also more likely not to use ART (aOR 4.56, 95% CI: 1.79–11.57, p = 0.001) when compared to non-drug users (Table 4).

**Table 4:** Multiple regression analysis showing associations between selected characteristics of HIV-infected individuals with ART non-use among individuals aged 15 to 64 years, South Africa, 2008 and 2012.

Variable	South Africa, 2008		South Africa, 2012	
	Multiple Regression Analysis n = 1170		Multiple Regression Analysis n = 2138	
	aOR (95% CI)	p-value	aOR (95% CI)	p-value
**Locality type**				
Urban formal (ref)	Ref		ref	
Urban informal	0.49 (0.24–0.98)	0.04	1.20 (0.75–1.92)	0.441
Rural formal	1.20 (0.38–0.82)	0.75	0.85 (0.46–1.54)	0.583
Rural informal	0.52 (0.25–1.09)	0.084	0.78 (0.50–1.20)	0.259
**Province**				
KwaZulu-Natal (ref)	ref			
Western Cape	0.40 (0.13–1.20)	0.104		
Eastern Cape	0.86 (0.38–1.97)	0.727		
Northern Cape	0.60 (0.20–1.80)	0.358		
Free State	1.49 (0.63–3.54)	0.365		
Northwest	0.71 (0.30–1.68)	0.431		
Gauteng	0.70 (0.30–1.62)	0.402		
Mpumalanga	0.84 (0.38–1.83)	0.656		
Limpopo	1.50 (0.49–4.55)	0.477		
**Sex**				
Male (ref)			ref	
Female			0.73 (0.52–1.04)	0.084
**Age (years)**				
15–24	2.68 (0.813–8.808)	0.105	4.23 (2.56–6.70)	<0.001
25–34	0.67 (0.30–1.49)	0.326	2.84 (1.73–4.67)	<0.001
35–49	0.55 (0.27–1.14)	0.108	1.08 (0.50–1.20)	0.741
50–64 (ref)	ref		ref	
**Marital status**				
Single/Never married/no stable partnership (ref)	ref		ref	
Married/stable partnership	1.52 (0.85–2.72)	0.159	1.25 (0.86–1.82)	0.241
Divorced/Separated/Widowed	0.45 (0.22–0.91)	0.027	0.99 (0.59–1.67	0.977
**Education**				
None (ref)	ref			
Primary	0.34 (0.97–1.22)	0.099		
Secondary	0.29 (0.09–0.99)	0.048		
Higher education	0.14 (0.03–0.66)	0.014		
**Employment**				
Unemployed (ref)	ref		ref	
Employed	2.16 (1.30–3.94)	0.004	2.53 (0.91–7.07)	0.075
Student	0.32 (0.06–1.76)	0.191	1.61 (1.16–2.23)	0.004
**Household wealth quintile**				
Quintile I (ref)	ref			
Quintile II	0.89 (0.47–1.69)	0.724		
Quintile III	0.443 (0.20–0.95)	0.037		
Quintile IV	0.82 (0.33–2.03)	0.673		
Quintile V	0.40 (0.13–1.26)	0.116		
**Alcohol intake in past 12 months**				
Non high-risk drinker (ref)			ref	
High-risk drinker			1.36 (0.72–2.55)	0.344
**Recreational drug use in past 3 months**				
No (ref)			ref	
Yes			4.56 (1.79–11.57)	0.001

## Discussion

Use of ART increased significantly in both countries, consistent with ART program scale-up and evolving treatment guidelines in both Kenya and South Africa during the study period. The proportion of PLHIV on ART was higher in Kenya than in South Africa both in 2007/2008 and 2012. However South Africa had a larger number of PLHIV compared to Kenya in both surveys, and the proportion of PLHIV on ART in South Africa nearly doubled over this period. While in South Africa it appears disparities in ART use may have decreased over the period of analysis, in Kenya household wealth among women and rural residency among men and women combined were both significantly associated with ART non-use in 2012 but not in 2007.

The analyses showed both similarities and differences in ART use in Kenya and South Africa. In both countries, after controlling for other covariates, there were no significant disparities in ART use by province and sex. In Kenya in 2012 lack of ART use was higher in rural areas while no differences were observed by locality type in South Africa in either survey year. Differences by household wealth were only seen in 2012 in Kenya, among women only, and in 2008 in South Africa, where they were limited to one quintile.

Older age was associated with greater likelihood of ART use in both countries in both survey periods when controlling for other factors. This may partially reflect natural disease progression and ART eligibility criteria at the time, with older people more likely to have been infected for a longer period of time compared to younger people, thus reaching the thresholds for ART treatment required by contemporary treatment guidelines. Nonetheless this finding of age differences in non-use of ART among PLHIV suggests need for strengthening efforts to ensure that younger people know their status, start ART, and adhere to care and treatment. In the 2012 KAIS 82% of HIV-infected youth aged 15–24 years (50.6%) were unaware of their HIV status, compared with 58.9% of those aged 25–34 [26], while the 2012 South Africa survey found a significantly lower proportion of youth aged 15–24 years had been tested for HIV compared with those aged 25–49 years (78.2%) [18]. With the adoption of the test-and-treat policy in 2016 in both countries, decreasing disparities in ART use by age group are expected in the future. Nonetheless, high uptake of testing among young people combined with appropriate linkage to care will be a necessary precondition to achieving adequate ART coverage. Future studies may also find that reduced ART use among women with a recent pregnancy, as seen in both survey years in Kenya, may have since been mitigated by changed eligibility criteria which made all pregnant women eligible for life-long ART with the adoption of option B+ for PMTCT starting in 2014 regardless of CD4 count in Kenya [21]. In South Africa this policy was implemented in January 2015 [27].

Overall, household wealth and educational level were not associated with ART use in either country in 2012, which is perhaps a result of the large, government-sponsored ART programs in both Kenya and South Africa offering ART to all regardless of ability to pay. However, in a sub-group analysis among women in Kenya, ART use was associated with increasing household wealth among women. In both countries, recent illicit/recreational drug use was associated with lower ART use, though drug users make up a small proportion of PLHIV. This finding could reflect low treatment access, adherence and/or retention in care in this population group. In a separate analysis of KAIS 2012, Mukui *et al*. found a significant association between both younger age and recreational drug use and poor adherence to treatment, and argue that increased identification and management of recreational drug use in this population is needed in order to mitigate its potential effects on adherence [28].

Kenya’s epidemic is highly geographically heterogeneous, with highest prevalence in the Nyanza region and the greatest burden of PLHIV in Nyanza and Nairobi [15]. Point estimates for ART use increased across all regions except Nyanza, though only Rift Valley increased significantly. Given that over a third of PLHIV in Kenya live in Nyanza, additional efforts to achieve increases in ART use in this region are warranted. The appearance of reduced ART use in rural relative to urban areas in 2012, not seen in 2007, may reflect either reduced barriers to access in urban areas or challenges in accessing drugs in rural areas due to increased travel costs, fear of stigma, or other barriers, and is a cause for concern [29,30].

In South Africa, HIV prevalence also varied across provinces and geographic locations, being highest in both urban and rural informal settlements in 2012. While ART use was lowest in rural informal settlements in 2008, it increased dramatically in 2012 reaching the same levels of use observed in other locality types, suggesting success in increasing geographical equity in ART access in South Africa over time. This suggestion is further corroborated by the absence of significant associations between ART non-use and province in both survey years and by locality type in 2012.

The lower levels of ART use among employed PLHIV versus unemployed in South Africa in 2008 might be due to a healthy worker effect, given that treatment initiation guidelines in South Africa historically recommended ART initiation based on CD4 count. Comparatively lower ART use among South African students in 2012, even when controlling for age, is an interesting finding, and could also be a reflection of the healthy worker effect as well as the natural disease progression as discussed above.

In Kenya, non-disclosure of HIV status to partners was significantly associated with higher adjusted odds of lack of ART use among PLHIV, suggesting that stigma may impact an individual’s decision regarding use of ART. The most recent stigma index survey conducted in Kenya in 2009–2010 found high levels of social exclusion (40%) and internalized stigma with 46% of PLHIV feeling guilty or ashamed for being HIV-positive [31]. In contrast, the stigma index survey conducted in South Africa in 2014 found low levels of social exclusion among PLHIV, however it revealed high levels of internalised stigma with 29% of participants in the survey reporting that they felt ashamed, while 28% had feelings of guilt [32]. This suggests need for continued stigma reduction strategies among PLHIV in both countries.

This analysis was subject to several limitations. Antiretroviral treatment access from 2007–2012 in both countries was highly dependent on treatment guidelines implemented at the time that individual patients engaged in care, and these guidelines have evolved over time. Our analysis did not attempt to establish eligibility for treatment, nor would it be entirely possible in a cross-sectional study to account for eligibility prior to ART initiation, but rather included all PLHIV regardless of eligibility. Since we measured presence of trace quantities of ART metabolites in blood we are not able to distinguish between PLHIV who have never been on ART, those who have stopped taking ART (i.e. disengagement from care) or those that have not taken their drugs for several days or weeks (i.e. non-adherent). Data regarding distance to the nearest facility offering ART, which may have provided further insights about ART accessibility, were not available. We also did not consider self-reported HIV status when assessing ART use. Finally, sample size was limited for some domains, especially in Kenya, which could have limited our power to detect differences in some cases and prevented more detailed sex-specific analyses.

In spite of these limitations, measurement of ART use in nationally-representative surveys in two countries with large HIV treatment programs allowed for an objective assessment of trends in ART use among PLHIV in the era of treatment expansion and identification of potential barriers to ART access and uptake. Both countries demonstrated substantial increases in ART use over time with broadly equitable access to ART across geographic and socioeconomic status with some exceptions. Our analyses did find evidence of increasing disparities in access to ART by household wealth in women in Kenya, and lingering inequities in use by age and among those who use recreational drugs, indicating some populations may require increased adherence support or access to HIV testing. To the degree that lower use in youth may be due to eligibility criteria, the adoption of test-and-treat provides a new opportunity to increase access to ART among youth.

## Supporting information

S1 Table Treatment guidelines by survey year in Kenya and South Africa. (DOCX)

S2 Table Comparison of observed and completed values for multiply-imputed variables, Kenya 2007 and 2012. (DOCX)

S3 Table Bivariate regression analysis showing associations between selected characteristics of HIV-infected individuals with exposure to ART among individuals 15–64 years, Kenya 2007 and 2012. (DOCX)

S4 Table Bivariate regression analysis showing associations between selected characteristics of PLHIV with non-use ART among individuals 15–64 years: South Africa 2008 and 2012. (DOCX)

S5 Table ARV exposure by selected characteristics of HIV-infected women aged 15–64 years old, Kenya 2007 and 2012. (DOCX)

S1 Text Statistical methods for addressing missing data. (DOCX)

S1 Fig Antiretroviral exposure by locality type among HIV-infected individuals aged 15–64 years old: South Africa, 2008 and 2012. (DOCX)
