# Supplementary material for: Equity of antiretroviral treatment use in high HIV burden countries: Analyses of data from nationally-representative surveys in Kenya and South Africa
Source: PLoS One. 2018 Aug 10;13(8):e0201899. doi: 10.1371/journal.pone.0201899 (PMC6086417; doi:10.1371/journal.pone.0201899)
Supplement: S1 Table — (DOCX) [file pone.0201899.s001.docx]

**S1 Table.** Treatment guidelines by survey year in Kenya and South Africa

| **Survey** | **Kenya** | **South Africa** |
| --- | --- | --- |
| **2007-2008** | *CD4<200 cells/mm^3^, or WHO stage III or IV* | *CD4<200 cells/mm^3^, WHO stage IV* |
| **2012** | *CD4<350 cells/mm^3^, or WHO stage III or IV, or co-infected with TB* | *CD4<200 cells/mm^3^, or WHO stage IV, or CD4<350 cells/mm^3^* *and pregnant or co-infected with TB* |
| **Present** | *Test-and-treat from mid-2016* | *Test-and-treat from 1^st^ September 2016* |
