# Supplementary material for: Equity of antiretroviral treatment use in high HIV burden countries: Analyses of data from nationally-representative surveys in Kenya and South Africa
Source: PLoS One. 2018 Aug 10;13(8):e0201899. doi: 10.1371/journal.pone.0201899 (PMC6086417; doi:10.1371/journal.pone.0201899)
Supplement: S2 Table — (DOCX) [file pone.0201899.s002.docx]

# S2 Table. Comparison of observed and completed values for multiply-imputed variables, Kenya 2007 and 2012

|  | **Kenya, 2007** | | | | **Kenya, 2012** | | | |
| --- | --- | --- | --- | --- | --- | --- | --- | --- |
|  | **observed data** | | **completed data** | | **observed data** | | **completed data** | |
| **Variable** | **Weighted %** | **95% CI** | **Weighted %** | **95% CI** | **Weighted %** | **95% CI** | **Weighted %** | **95% CI** |
| **ART status** |  |  |  |  |  |  |  |  |
| not on ART | 70.7 | (63.5-76.9) | 72.5 | (68-77) | 57.5 | (52.3-62.5) | 58.5 | (53.8-63.3) |
| on ART | 29.3 | (23.1-36.5) | 27.5 | (23-32) | 42.5 | (37.5-47.7) | 41.5 | (36.7-46.2) |
| **Current CD4 category (cells/mm^3^)** |  |  |  |  |  |  |  |  |
| <250 | 18.8 | (16.1-22) | 19.0 | (15.9-21.7) | 16.2 | (11.9-21.7) | 16.2 | (11.3-21.1) |
| 250>-350 | 12.5 | (10.3-15.1) | 12.4 | (10-14.8) | 14.8 | (10.4-20.6) | 14.8 | (9.7-19.9) |
| 350>-500 | 16.9 | (14.6-19.4) | 17.0 | (14.5-19.5) | 14.7 | (10.5-20) | 14.7 | (9.9-19.4) |
| >500 | 51.7 | (47.9-55.5) | 51.8 | (48-55.6) | 54.3 | (47-61.4) | 54.3 | (47.1-61.6) |
| **Disclosed results to most recent partner** |  |  |  |  |  |  |  |  |
| Yes | 77.6 | (75.9-79.2) | 77.3 | (75.6-79) | 21.4 | (18.2-25.1) | 21.5 | (18.1-24.9) |
| No | 9.8 | (8.6-11.1) | 9.7 | (8.5-11) | 49.5 | (44.6-54.4) | 49.3 | (44.4-54.1) |
| N/A | 12.6 | (11.4-14) | 13.0 | (11.6-14.3) | 29.1 | (25.1-33.5) | 29.2 | (25-33.5) |
