# Supplementary material for: Equity of antiretroviral treatment use in high HIV burden countries: Analyses of data from nationally-representative surveys in Kenya and South Africa
Source: PLoS One. 2018 Aug 10;13(8):e0201899. doi: 10.1371/journal.pone.0201899 (PMC6086417; doi:10.1371/journal.pone.0201899)
Supplement: S3 Table — (DOCX) [file pone.0201899.s003.docx]

# S3 Table. Bivariate regression analysis showing associations between selected characteristics of HIV-infected individuals with exposure to ART among individuals 15–64 years, Kenya 2007 and 2012

|  | **Kenya, 2007** | | | **Kenya, 2012** | | |
| --- | --- | --- | --- | --- | --- | --- |
| **Variable** | **OR** | **(95% CI)** | **p-value** | **OR** | **(95% CI)** | **p-value** |
| **Residency type** |  |  |  |  |  | 0.232 |
| Urban (ref) | ref |  |  | ref |  |  |
| Rural | 0.81 | (0.45-1.46) | 0.474 | 1.27 | (0.86-1.89) |  |
| **Province** |  |  | 0.518 |  |  | 0.648 |
| Central | 0.67 | (0.27-1.67) |  | 0.83 | (0.4-1.7) |  |
| Coast | 1.71 | (0.63-4.68) |  | 1.10 | (0.47-2.59) |  |
| Eastern | 1.02 | (0.36-2.88) |  | 0.63 | (0.27-1.49) |  |
| Nairobi (ref) | ref |  |  | ref |  |  |
| Nyanza | 0.80 | (0.34-1.87) |  | 1.18 | (0.65-2.14) |  |
| Rift Valley | 1.26 | (0.48-3.31) |  | 0.94 | (0.42-2.09) |  |
| Western | 0.84 | (0.33-2.16) |  | 1.31 | (0.61-2.82) |  |
| **Female sex** | 0.92 | (0.61-1.38) | 0.673 | 0.80 | (0.54-1.2) | 0.288 |
| **Age (years)** |  |  | 0.002 |  |  | <0.001 |
| 15-24 | 3.69 | (1.55-8.79) |  | 5.60 | (2.45-12.78) |  |
| 25-34 | 2.18 | (0.96-4.95) |  | 4.09 | (2.4-6.96) |  |
| 35-49 | 1.31 | (0.68-2.51) |  | 1.22 | (0.72-2.05) |  |
| 50-64 (ref) | ref |  |  | ref |  |  |
| **Marital status** | | | 0.069 |  |  | <0.001 |
| single/never married (ref) | ref |  |  | ref |  |  |
| married/cohabitating | 0.67 | (0.33-1.38) |  | 0.38 | (0.21-0.71) |  |
| divorced/separated/widowed | 0.44 | (0.2-1) |  | 0.27 | (0.14-0.53) |  |
| **Education** |  |  | 0.921 |  |  | 0.190 |
| None (ref) | ref |  |  | ref |  |  |
| Primary | 1.04 | (0.62-1.75) |  | 0.88 | (0.5-1.56) |  |
| Secondary | 0.87 | (0.45-1.69) |  | 0.76 | (0.29-1.97) |  |
| Higher | 0.80 | (0.28-2.28) |  | 0.57 | (0.32-1.04) |  |
| **Employment status** |  |  |  |  |  | 0.078 |
| Employed | 1.04 | (0.59-1.84) | 0.880 | 1.41 | (0.96-2.07) |  |
| Unemployed (ref) | ref |  |  | ref |  |  |
| **Household wealth quintile** |  |  | 0.736 |  |  | 0.934 |
| Quintile I (ref) | ref |  |  | ref |  |  |
| Quintile II | 1.07 | (0.54-2.11) |  | 0.95 | (0.46-1.95) |  |
| Quintile II | 0.74 | (0.36-1.52) |  | 0.93 | (0.46-1.86) |  |
| Quintile IV | 0.75 | (0.35-1.58) |  | 0.88 | (0.45-1.73) |  |
| Quintile V | 0.76 | (0.38-1.52) |  | 0.75 | (0.36-1.55) |  |
| **CD4 category (cells/mm^3^)** |  |  | 0.010 |  |  | 0.301 |
| <250 (ref) | ref |  |  | ref |  |  |
| 250-349 | 0.40 | (0.19-0.87) |  | 0.63 | (0.25-1.59) |  |
| 350-499 | 0.74 | (0.37-1.47) |  | 1.16 | (0.45-3.02) |  |
| 500+ | 1.23 | (0.7-2.17) |  | 1.30 | (0.65-2.59) |  |
| **Testing history** |  |  | 0.675 | 2.08 |  | 0.013 |
| <1 year ago (ref) | ref |  |  | ref |  |  |
| 1-2 years ago | 0.80 | (0.42-1.51) |  | 1.93 | (1.16-3.22) |  |
| >2 years ago | 0.77 | (0.41-1.45) |  | 0.85 | (0.52-1.37) |  |
| **Disclosure of HIV status to most recent partner*** | | |  |  |  | 0.011 |
| No | 1.60 | (0.55-4.65) | 0.381 | 2.08 | (1.18-3.66) |  |
| Yes (ref) | ref |  |  | ref |  |  |
| Pregnant in last 3 years † | 1.77 | (1.05-2.97) | 0.033 | 1.66 | (1.01-2.73) | 0.045 |
| ANC visit last pregnancy † | 0.78 | (0.17-3.66) | 0.752 | 1.02 | (0.06-16.89) | 0.991 |
| Any HIV test during ANC last pregnancy † | 0.63 | (0.24-1.67) | 0.350 | 2.08 | (0.36-11.96) | 0.405 |
| **Sex and pregnancy history** | |  | 0.078 |  |  | 0.098 |
| Male (ref) | ref |  |  | ref |  |  |
| Female - not pregnant in last 3 years | 0.76 | (0.48-1.22) |  | 0.71 | (0.46-1.1) |  |
| Female - pregnant in last 3 years | 1.35 | (0.82-2.22) |  | 1.19 | (0.71-1.99) |  |
| **Recreational drug use in last 12 months** |  |  |  |  |  | 0.003 |
| No (ref) | ref |  |  | ref |  |  |
| Yes | - | - | - | 3.22 | (1.51-6.87) |  |

Notes: * Applies to respondents who self-reported HIV-positive. † Applies to women only.
