# Supplementary material for: Equity of antiretroviral treatment use in high HIV burden countries: Analyses of data from nationally-representative surveys in Kenya and South Africa
Source: PLoS One. 2018 Aug 10;13(8):e0201899. doi: 10.1371/journal.pone.0201899 (PMC6086417; doi:10.1371/journal.pone.0201899)
Supplement: S4 Table — (DOCX) [file pone.0201899.s004.docx]

**S4 Table.** Bivariate regression analysis showing associations between selected characteristics of PLHIV with non-use ART among individuals 15–64 years: South Africa 2008 and 2012

|  | **South Africa, 2008** | | **South Africa, 2012** | |  |
| --- | --- | --- | --- | --- | --- |
|  | **Bivariate Analysis** | | **Bivariate Analysis** | |  |
| **Variable** | **OR (95% CI)** | **p-value** | **OR (95% CI)** | **p-value** | |
| **Locality type** |  |  |  |  | |
| Urban formal (ref) | ref |  | ref |  | |
| Urban informal | 0.73 (0.40-1.34) | 0.313 | 0.99 (0.67-1.48) | 972 | |
| Rural formal | 0.79 (0.45-1.38) | 0.405 | 0.98 (0.57-0.69) | 0.935 | |
| Rural informal | 2.25 (0.94-5.42) | 0.069 | 0.70 (0.49-1.00) | 0.053 | |
| Province |  |  |  |  | |
| KwaZulu-Natal (ref) | ref |  | ref |  | |
| Western Cape | 2.16 (0.91-5.12) | 0.079 | 1.29 (0.74-2.23) | 0.372 | |
| Eastern Cape | 1.67 (0.59-4.69) | 0.333 | 1.20 (0.66-2.17) | 0.549 | |
| Northern Cape | 1.69 (0.55-5.21) | 0.364 | 0.42 (0.138-1.25) | 0.119 | |
| Free State | 2.53(1.01-6.35) | 0.048 | 0.98 (0.52-1.84) | 0.938 | |
| Northwest | 1.44 (0.59-3.52) | 0.423 | 1.73 (0.89-3.36) | 0.105 | |
| Gauteng | 1.72 (0.65-4.53) | 0.27 | 0.94 (0.47-1.88) | 0.853 | |
| Mpumalanga | 2.01 (0.84-4.80) | 0.115 | 1.48 (0.79-2.76) | 0.223 | |
| Limpopo | 2.45 (0.89-6.77) | 0.083 | 0.87 (0.40-1.88) | 0.722 | |
| **Race** |  |  |  |  | |
| Other race (ref) | ref |  | ref |  | |
| Black African | 0.93 (0.38-2.26) | 0.864 | 1.61 (0.91-2.85) | 0.102 | |
| **Sex** |  |  |  |  | |
| Male (ref) | ref |  | ref |  | |
| Female | 1.13 (0.67-1.88)) | 0.65 | 0.61 (0.46-0.81) | 0.001 | |
| **Age (years)** |  |  |  |  | |
| 15-24 | 1.33 (0.44-4.02) | 0.612 | 4.57 (2.70-7.22) | <0.001 | |
| 25-34 | 0.81 (0.41-1.62) | 0.556 | 2.75 (1.85-4.08) | <0.001 | |
| 35-49 | 0.67 (0.34-1.29) | 0.228 | 1.10 (0.75-1.60) | 0.63 | |
| 50-64 (ref) | ref |  | ref |  | |
| Marital status |  |  |  |  | |
| Single/Never married/no stable partnership (ref) | ref |  | ref |  | |
| Married/stable partnership | 1.39 (0.83-2.34) | 0.208 | 1.30 (0.90-1.89) | 0.161 | |
| Divorced/Separated/Widowed | 0.53 (0.28-1.00) | 0.05 | 0.56 (0.35-0.91) | 0.017 | |
| **Education** |  |  |  |  | |
| None (ref – 2008) | ref |  | n/a | n/a | |
| Primary (ref – 2012) | 0.36 (0.11-1.23) | 0.103 | ref |  | |
| Secondary | 0.33 (0.10-1.04) | 0.059 | 1.12 (0.84-1.48) | 0.441 | |
| Higher education | 0.15 (0.03-0.76) | 0.021 | 1.36 (0.57-3.25) | 0.493 | |
| **Employment** |  |  |  |  | |
| Unemployed (ref) | ref |  | ref |  | |
| Employed | 2.00 (1.26-3.18) | 0.003 | 4.79 (1.93-11.88) | 0.001 | |
| Student | 0.90 (0.17-4.75) | 0.901 | 1.33 (1.02- 1.74) | 0.033 | |
| **Household wealth quintile** |  |  |  |  | |
| Quintile I (ref) | ref |  | ref |  | |
| Quintile II | 0.99 (0.57-1.74) | 0.983 | 1.13 (0.82-1.55) | 0.467 | |
| Quintile III | 0.52 (0.27-0.98) | 0.042 | 1.02 (0.68-1.53) | 0.932 | |
| Quintile IV | 1.23 (0.59-2.56) | 0.555 | 1.26 (0.79-2.02) | 0.334 | |
| Quintile V | 0.58 (0.18-1.84) |  | 0.76 (0.38-1.50) | 0.426 | |
| **Most recent HIV test** |  |  |  |  | |
| <12 months (ref) | ref |  | ref |  | |
| 1-2 years | 1.32 (0.68-2.58) | 0.406 | 0.09 (0.71-1.67) | 0.69 | |
| 2-3 years | 0.91 (0.41-2.05) | 0.822 | 1.24 (0.77-2.01) | 0.365 | |
| 3+ years | 0.61 (0.29-1.29) | 0.197 | 0.69 (0.44-1.08) | 0.103 | |
| **Alcohol intake in the last 12 month** |  | |  |  | |
| Non high-risk drinker (ref) | ref |  | ref |  | |
| High-risk drinker | 0.75 (0.34-1.68) | 0.492 | 1.71 (0.96-3.04) | 0.069 | |
| **Recreational drug use in the last 3 months** |  |  |  |  | |
| No (ref) | ref |  | ref |  | |
| Yes |  |  | 4.80 (2.20-10.47) | <0.001 | |
