# Supplementary material for: Equity of antiretroviral treatment use in high HIV burden countries: Analyses of data from nationally-representative surveys in Kenya and South Africa
Source: PLoS One. 2018 Aug 10;13(8):e0201899. doi: 10.1371/journal.pone.0201899 (PMC6086417; doi:10.1371/journal.pone.0201899)
Supplement: S5 Table — (DOCX) [file pone.0201899.s005.docx]

# S5 Table. ARV exposure by selected characteristics of HIV-infected women aged 15–64 years old, Kenya 2007 and 2012

|  | **Kenya, 2007** | | | **Kenya, 2012** | | |
| --- | --- | --- | --- | --- | --- | --- |
|  | **On ART** | | | **On ART** | | |
| **Variable** | **N** | **Weighted % (95% CI)** | **p-value** | **N** | **Weighted % (95% CI)** | **p-value** |
| **Locality type** |  |  | 0.715 |  |  | 0.576 |
| Rural | 255 | 30.1 (21.0-39.3) |  | 216 | 42.7 (35.6-49.8) |  |
| Urban | 117 | 33.7 (16.6-50.7) |  | 168 | 49.3 (42.0-56.6) |  |
| **Province** |  |  | 0.271 |  |  | 0.721 |
| Nairobi | 46 | 40.2 (10.0-70.5) |  | 37 | 49.7 (37.1-62.2) |  |
| Central | 23 | * |  | 40 | 51.4 (36.8-66.0) |  |
| Coast | 49 | 12.4 (1.5-23.2) |  | 40 | 38.6 (20.8-56.3) |  |
| Eastern | 35 | 39.2 (10.5-67.9) |  | 53 | 69.2 (50.3-88.0) |  |
| Nyanza | 135 | 35.5 (20.6-50.4) |  | 132 | 40.7 (33.8-47.5) |  |
| Rift Valley | 48 | 18.3 (7.4-29.2) |  | 45 | 40.8 (25.1-56.5) |  |
| Western | 36 | 37.1 (13.0-61.2) |  | 37 | 40.5 (28.8-52.2) |  |
| **Age (years)** |  |  | <.001 |  |  | 0.007 |
| 15-24 | 83 | 18.2 (6.9-29.5) |  | 50 | 17.7 (7.4-28.0) |  |
| 25-34 | 136 | 24.3 (14.0-34.6) |  | 123 | 29.6 (21.1-38.1) |  |
| 35-49 | 126 | 43.5 (32.2-54.9) |  | 157 | 59.1 (51.2-67.0) |  |
| 50-64 | 27 | 38.9 (21.5-56.3) |  | 54 | 67 (53.4-80.6) |  |
| **Marital status** |  |  | <.001 |  |  | 0.822 |
| Single/never married | 42 | 25.8 (9.0-42.6) |  | 51 | 19.4 (9.3-29.6) |  |
| Married/cohabitating | 220 | 23.7 (16.3-31.1) |  | 201 | 46 (39.1-52.8) |  |
| Divorced/separated/widowed | 110 | 45.8 (33.6-58.0) |  | 132 | 54.6 (45.7-63.6) |  |
| **Education** |  |  | 0.394 |  |  | 0.937 |
| None | 212 | 34.3 (23.1-45.5) |  | 64 | 37.6 (22.7-52.5) |  |
| Primary | 111 | 24.7 (15.9-33.5) |  | 187 | 40.1 (33.3-47.0) |  |
| Secondary | 36 | 32.3 (15.2-49.4) |  | 18 | * |  |
| Higher | 13 | * |  | 114 | 56.2 (46.8-65.6) |  |
| **Household wealth** |  |  | 0.815 |  |  | 0.886 |
| Quintile I (lowest) | 71 | 30.4 (16.2-44.6) |  | 61 | 37.9 (23.5-52.2) |  |
| Quintile II | 72 | 24.4 (11.5-37.3) |  | 89 | 42.4 (31.1-53.8) |  |
| Quintile III | 58 | 34.2 (18.7-49.7) |  | 86 | 44.5 (32.2-56.8) |  |
| Quintile IV | 81 | 33.1 (17.3-48.9) |  | 92 | 49.8 (38.5-61.1) |  |
| Quintile V (highest) | 90 | 34 (21.9-46.1) |  | 56 | 54 (41.8-66.2) |  |
| **Employment** |  |  | 0.646 |  |  | 0.019 |
| Unemployed | 81 | 33.8 (18.7-49.0) |  | 165 | 47.8 (39.3-56.3) |  |
| Employed | 291 | 30.4 (22.3-38.5) |  | 218 | 44.2 (37.7-50.7) |  |
| **CD4 category (cells/mm^3^)** |  |  | <.001 |  |  | 0.086 |
| <=250 | 58 | 20.5 (8.8-32.2) |  | 29 | 46.7 (29.0-64.3) |  |
| 250>-350 | 45 | 58.5 (42.5-74.5) |  | 25 | 60.3 (40.0-80.5) |  |
| 350>-500 | 56 | 34.2 (20.7-47.6) |  | 24 | * |  |
| >500 | 184 | 27 (17.1-36.9) |  | 120 | 48 (38.3-57.7) |  |
| **Most recent HIV test †** |  |  | 0.897 |  |  | 0.690 |
| <12 months ago | 92 | 40.7 (28.4-53.0) |  | 188 | 51 (42.5-59.6) |  |
| 1-2 years ago | 55 | 44.6 (28.0-61.1) |  | 70 | 29.4 (18.3-40.5) |  |
| > 2 years ago | 56 | 41.6 (25.9-57.4) |  | 89 | 55.9 (45.7-66.0) |  |
| **Disclosed results to most recent partner** ‡ |  |  | 0.369 |  |  | <0.001 |
| Yes | 135 | 31.2 (21.1-41.3) |  | 78 | 61.9 (49.3-74.4) |  |
| No | 19 | * |  | 126 | 65.4 (56.9-73.9) |  |
| **Recreational drug use in last 12 months** |  |  |  |  |  | 0.002 |
| Yes | - |  |  | 7/19 | * |  |
| No |  |  |  | 365 | 46.2 (40.9-51.4) |  |
| **Total** | 372 | 31.2 (23.0-39.4) |  | 384 | 45.6 (40.5-50.7) |  |

Notes: All numbers are unweighted except where otherwise specified. * Suppressed due to denominator <25 observations. **†** Applies to respondents who reported ever testing for HIV ‡ Applies to respondents who self-reported HIV-positive. ART = antiretroviral treatment, CI = confidence interval.
