# Supplementary material for: Equity of antiretroviral treatment use in high HIV burden countries: Analyses of data from nationally-representative surveys in Kenya and South Africa
Source: PLoS One. 2018 Aug 10;13(8):e0201899. doi: 10.1371/journal.pone.0201899 (PMC6086417; doi:10.1371/journal.pone.0201899)
Supplement: S1 Text — (DOCX) [file pone.0201899.s006.docx]

# S1 Text. Statistical Methods for addressing missing data

In order to address missing values for several key variables including ART use (the dependent variable) and CD4 count, we used multiple imputation. Potential targets for imputation were selected by first inspecting patterns of missing values using the Stata mi misstable command, resulting in the selection of ART use (the dependent variable), CD4 count, and disclosure of HIV status to sex partner as targets of imputation. We assumed that missing values were missing at random (MAR), in other words that the missingness can be completely explained by other observed covariates in the dataset. Given that missing ART status and CD4 count is generally due to laboratory transport or processing errors, or due to depletion of sample, all of which generally occur independently from factors likely to influence ART use, CD4 count or disclosure of HIV status, this assumption seemed plausible. We used multiple imputation with chained equations (MICE), implemented with the Stata mi command. This method was selected due to the ready availability of implementations in statistical software, as well as its flexibility and simplicity of specifying restrictions on imputed values (for example, allowing the exclusion of specific auxiliary variables in the model for specific imputation targets). Imputations were run separately on each survey. First, variables identified as likely to be associated with the outcome of interest were selected. Additional auxiliary variables were also selected. In the case of 2007, the following variables were selected as targets for multiple imputation based on the proportion of responses that were missing and their relevance to the primary analysis: CD4 category, ART status, and disclosure of HIV status to most recent sex partner. The following variables were selected as predictors of the response variables: cluster number, province, sex, wealth index quintile, employment status, educational level, age group, marital status, locality, history of HIV testing. Self-reported ART status was considered for inclusion but dropped due to colinearity with the dependent variable, as were household-level variables. The data were svyset before analysis using the normalized KAIS blood weights. The following equation was used to create 20 imputed datasets for the 2007 KAIS for HIV-positive respondents using chained equations to allow multiple outcomes to be imputed sequentially.

The imputation was restricted to HIV-positive respondents, as only these respondents are included in the analysis of ART use. CD4 category is an ordinal variable so was imputed using an ordered logistic model, ART status is a Boolean variable so was imputed using a logistic model, while disclosure status was a categorical variable with three unordered levels and was imputed using multinomial logistic regression. The MICE method assumes a joint normal distribution of imputed values.

After imputation, 517 of 522 missing ART status had been imputed, 81 of 84 missing CD4 category had been imputed, and 61 of 61 missing disclosure had been imputed. Not all cases were successfully imputed due to missingness of the predictors.

For the 2012 KAIS, CD4 count was not included as a target for imputation as the proportion of missing values was low. Cluster number was omitted from the predictors due to colinearity with ART status. Self-reported ART status was also included as a predictor of disclosure, but omitted from predicting the ART biomarker again due to colinearity. Several household-level variables were also found to be predictive of the response and were included in the final equation: sex of head of household, whether head of household was known to be HIV-infected, HIV test result of head of household, and the household hunger scale index.

After imputation, of 648 HIV-positive respondents, 89 missing ART status had been imputed, while 20 missing disclosure status had been imputed (there were no cases where imputation was unsuccessful).

To assess imputation errors such as misspecification of the imputation model or convergence issues, trace plots were used to look for patterns in the imputations to assess possible trends in imputed values or variances. The Stata mi xeq command was used to inspect and compare distributions of imputed values for individual imputations. Supplemental Table S2 compares the distribution of values for the observed and complete datasets.
