# Supplementary material for: Equity of antiretroviral treatment use in high HIV burden countries: Analyses of data from nationally-representative surveys in Kenya and South Africa
Source: PLoS One. 2018 Aug 10;13(8):e0201899. doi: 10.1371/journal.pone.0201899 (PMC6086417; doi:10.1371/journal.pone.0201899)

# S1 Fig. Antiretroviral exposure by locality type among HIV-infected individuals aged 15–64 years old: South Africa, 2008 and 2012


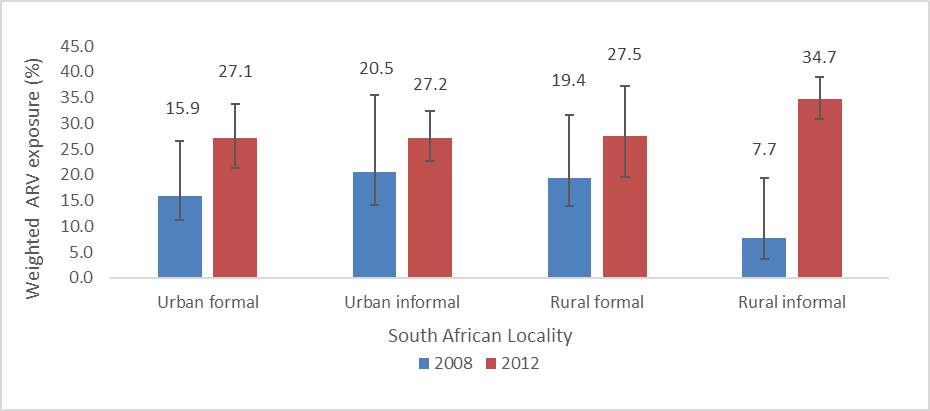

Supplement: S1 Fig — (DOCX) [file pone.0201899.s007.docx]
